# Creation, dissemination, and evaluation of videos to promote COVID-19 vaccination in India: A research protocol

**DOI:** 10.12688/gatesopenres.13628.1

**Published:** 2022-07-25

**Authors:** Ananya Bhaktaram, Rohini Ganjoo, Amelia M. Jamison, Julia Burleson, Paola Pascual-Ferra, Neil Alperstein, Daniel J. Barnett, Satyanarayan Mohanty, Peter Z. Orton, Manoj Parida, Eleanor Kluegel, Sidharth Rath, Rajiv N. Rimal

**Affiliations:** 1 Health, Behavior and Society, Johns Hopkins Bloomberg School of Public Health, Baltimore, Maryland, 21205, USA; 2 Biomedical Laboratory Sciences, George Washington University, Washington, D.C, 20052, USA; 3 Department of Communication, Loyola University of Maryland, Baltimore, MD, 21210, USA; 4 Environmental Health and Engineering, Johns Hopkins Bloomberg School of Public Health, Baltimore, Maryland, 21205, USA; 5 D-Cor (Development Corner) Consulting Pvt. Ltd, Satya Nagar, Bhubaneswar, Odisha, 751008, India; 6 Wellflix Inc, Hillsborough, NC, 27278, USA; 7 Swasthya Plus, Odisha, India, Chandrasekharpur, Bhubaneswar, Odisha, 751017, India

**Keywords:** Vaccination, COVID-19, entertainment-education videos, humor, gender, collectivist, vaccine hesitancy, messaging.

## Abstract

**Background:** Vaccine hesitancy is one of the greatest challenges to the success of coronavirus disease 2019 (COVID-19) vaccination campaigns. Videos promoting vaccines have a narrow scope focusing solely on facts, and less on the emotional and narrative elements of communication that can be equally persuasive. The role of humor, for example, has remained largely unexplored.

**Objective:** This study investigates whether theory-based videos can change people’s attitudes, beliefs, and intentions to receive the second COVID-19 vaccine. Our primary research question is: How do collectivistic and individualistic appeals, humor, and protagonist gender individually and jointly affect vaccination attitudes, beliefs, and intentions?

**Methods:** This project tapped into the underutilized Indian film industry—the world’s largest film producer—to promote vaccination messaging through short videos. Feedback from a community advisory board was utilized to inform the video scripts that were then shot by a production team. Eight videos were filmed and shared by adopting a 2 (appeal: individualistic or collectivistic) x 2 (tone: humor or non-humor) x 2 (protagonist gender: male or female) between-subjects design approach. Our sample includes Odia-speaking participants aged between 18 – 35 years old randomly assigned to watch one of the eight study videos. An online survey questionnaire, social media network analysis, and small group qualitative interviews will be utilized to explore how the entertainment-education videos can be used to reduce vaccine hesitancy.

**Discussion:** Vaccine messages do not fall into a cultural or cognitive vacuum. People process and make sense of information based on their prior experience, properties of the message, and their social environment. Yet, these considerations have taken secondary importance in vaccine communications. This research shows that it is possible to deliver high-caliber videos created in accordance with the audience's cultural and cognitive background.

**Conclusions: **This study will inform future health promotion messaging through brief videos on the internet.

## Introduction

As the coronavirus disease 2019 (COVID-19) pandemic enters its third year, more than 315 million cases have been recorded and more than 5.5 million people have died worldwide ^1^. India has experienced the second-largest burden of COVID-19, with more than 40 million confirmed cases and nearly half a million cumulative deaths ^2^. Although the country was largely spared during the initial outbreak, a second wave' of the virus swept the country in spring 2021 ^3^. With this surge, experts believe official statistics may have undercounted the true burden of the disease with a preliminary analysis of mortality rates suggesting as many as 3.97 million deaths occurring since the start of the pandemic ^4^.

India’s COVID-19 vaccination campaign was launched in January 2021 with an ambitious goal of fully immunizing all 1.3 billion Indian citizens against COVID-19 by the end of the year ^5^. The size of the population makes this campaign the world’s largest vaccination program ^6^. Initially, two vaccine products were authorized for use: Covishield produced by AstraZeneca and the locally-produced Covaxin, both requiring two doses administered 4 weeks (Covaxin) and 12-16 weeks (Covishield) apart ^7^. By the end of 2021, more than 1.5 billion doses of the COVID-19 vaccine had been administered, with an estimated 90% of the adult population having received one dose ^5^. However, among the vaccinated, roughly 1 in 3 had not received a second shot and were not fully protected ^8^. In addition, the demand for vaccines has declined since November 2021, the number of daily doses administered has tapered, with officials underscoring that decreased public demand as opposed to supply issues are driving the slowdown ^5^.

## Vaccine hesitancy

Globally, COVID-19 vaccination campaigns have been hindered by rising vaccine hesitancy, defined by the World Health Organization (WHO) as a “delay in acceptance or refusal of vaccination despite availability of vaccination services” ^9^. The WHO also describes major components that influence hesitancy, including: complacency, or the perceived need for vaccination; convenience, or the ease of obtaining a vaccine; and confidence, or the trust in the vaccine and the systems that produce and recommend it ^9^. Importantly, vaccine hesitancy is context specific, varying by population, region, and over time ^9^. In this conceptualization, instead of a binary defined by behavior, vaccine behavior and attitudes fall along a continuum, from active demand and acceptance to complete refusal ^10^. For instance, hesitant individuals may accept a vaccine but still harbor concerns, or intend to get a vaccine but delay initiation, while others may start a vaccine series but fail to complete all required doses. Although the dynamics of vaccine hesitancy in North America, Western Europe, and Australia have been well studied, comparatively little research has focused on vaccine hesitancy in Southeast Asia in general and India specifically ^11, 12^.

Pre-pandemic, India’s suboptimal vaccination rates were more often attributed to barriers to access, and not hesitancy ^11^. Recent examples can be tied to specific vaccines in discrete (and often marginalized) communities, including religious minorities, those living in slums, tribal populations, and those from scheduled castes ^11, 13, 14^. For instance, in the early 2000’s Larson *et al*. documented resistance to polio vaccine campaigns led by international NGOs as rumors of vaccine-induced sterilization resonated with Muslim communities in a majority Hindu state ^15^. Unlike in the West, these isolated events have not coalesced into a recognizable “anti-vaccine” movement ^11^. Generally, vaccine acceptance has been high and the successes of the national Universal Immunization Program (UIP) in eliminating vaccine preventable childhood diseases has led to positive sentiment towards vaccines. It is important to note that prior to COVID-19 vaccines, no vaccines were authorized for universal administration to Indian adults, and available research on vaccine hesitancy is restricted mostly to parental hesitancy toward childhood vaccines ^16^. Further, given the large, dense population in India, even a small increase in vaccine hesitancy could result in a large unvaccinated population.

The COVID-19 pandemic along with the introduction of novel vaccines has also been linked to an “infodemic” where a deluge of health claims -- including those that are unverified, misleading, and outright false -- have complicated the health communication environment ^17^. Exposure to misinformation around COVID-19 and COVID-19 vaccines contributes to vaccine hesitancy, particularly as social media use has increased ^18^. Social media also allowed organized international anti-vaccine groups from the US and elsewhere to reach receptive Indian audiences ^19^. Access to multiple public platforms like Twitter and Facebook, and private or encrypted platforms like WhatsApp, has complicated researchers’ ability to effectively assess the impacts of vaccine misinformation ^20^. Indeed, little is known about the reasons why there is a hesitancy gap between the first and second vaccine doses.

Empirical data focused on Odisha, an Eastern state in India and our study setting, is limited. A 2020 survey relying on a small convenience sample (<400 people) by Panda *et al*. (2021) found high confidence in COVID-19 vaccines and support for mandatory vaccination, but also found that respondents believed immunity from a naturally occurring infection would be more effective than vaccine-induced immunity ^21^. Data from the COVID-19 Symptom Survey suggests that early in the vaccination rollout, hesitancy was lower in Odisha than in many other Indian States, with less than 25% of respondents reporting unwillingness to get vaccinated ^6^. Traditionally, studies on vaccine communication have focused on several key communication elements such as the extent to which messages resonate with cultural values, how the messages are communicated (the appeal), and the source of the communication. However, vaccine hesitancy is context specific and has not been widely studied in India. Before structural and access-related issues can be addressed, we must first explore the social norms, influences and dynamics of vaccine hesitancy and complacency within India to see how they differ from the vaccine hesitancy literature that focus predominantly on North America and Western Europe. Thus, this paper focuses on social norms and cultural attitudes while exploring specifically how individualism-collectivism as the cultural factor; humor versus seriousness as the appeal; and gender as the key source characteristic are important to understand vaccine hesitancy.

## Role of humor

Health communication professionals designing vaccine promotion campaigns have to make decisions regarding the overall tone to adopt with campaigns focusing more on presenting facts and information, and less on the emotional and narrative elements of communication ^22^. The use of humor, in particular, has been largely overlooked.

Several studies have examined the use of humor by Indian youth as a coping mechanism, to navigate sensitive cultural normativity topics, and to enhance interpersonal relationships ^23, 24^. According to Hiranandani and Bing Yue (2014), humor is emphasized as an important element in Indian societies. In their study of humor styles among Indian university students, they found that the students use humor (‘mazaak’) to enhance social harmony, and that humor is “their preferred social interaction style and social influence of significant others” (p. 3). Hall (2019) indicates how mobile phones have been routinely used to spread jokes among hundreds of thousands of Indian mobile users. YouTube has been a primary platform for cultural expression, creativity, innovation and is a popular platform among Indian comedians ^25– 28^. In the study by Chaturvedi (2019), humor is one of the different elements used in a public health campaign in India aimed at mitigating the stigma of mental health through a series of YouTube videos ^29^. Ample research points to humor’s ability to gain attention, boost social influence, improve advertising and public relations campaigns, and facilitate strong communication between parties ^30, 31^. Moreover, in public service announcements (PSAs) and preparedness campaigns, humorous messages demonstrate an ability to enhance audiences’ attention and reduce counter-arguing through a light-hearted and likeable delivery of information ^30^. However, there is also the potential that humor may trivialize or otherwise hurt the perceived severity and credibility of the information being communicated, which may reduce an audiences’ intentions or likelihood to act on the information provided ^30^. For audiences in India specifically, humor has been credited in its ability to evoke an empathetic and understanding response in viewers ^29^. The few vaccine campaigns with a humorous twist are being appreciated and are popular on social media ^32, 33^.

 Observational studies from the COVID-19 pandemic also support this idea, with several content analyses of trending TikTok videos finding that humor is a dominant feature of content related to COVID-19 vaccines ^34– 36^. At the same time, humor has also been used to promote COVID-19 vaccine misinformation on the same platforms ^35^. There is considerable research complicating and contradicting the positive role of humor in health communication. Meyer and Venette explain that while potentially more memorable, using humor in health risk messages may mask or jeopardize the communication of serious health information, undermine the seriousness of the information, source credibility, and polarize or divide the audience ^31, 37^. In addition to the appropriateness and extent of humor used, different types of humor may differentially influence message perception. For example, Iles and Nan investigate the differences between sarcastic and ironic humor and found that both messaging styles reduced persuasiveness and increased counter-arguing—opposing earlier findings ^30, 37, 38^. The challenge for health communicators is not only the choice of tone (factual vs. humorous) but even more complex—what kind of fact(s) and what kind of humor makes for effective messaging for a specific context and audience.

## Role of individualism and collectivism

Adding another layer of complexity, socio-cultural norms, such as individualism and collectivism, heavily influences how people conduct their lives and describes the degree of social cohesion in a society and an individual’s willingness to prioritize common goals over personal goals ^39, 40^. In India, collectivist and individualist values coexist and are also reflected in people's behavior ^41^. For example, some young Indians may move to urban centers for work and have an individualistic lifestyle there but send a large portion of their income to their families in rural areas demonstrating collectivism ^41^. Many studies have characterized India as a more collectivist-leaning society highly valuing an emotionally close, well-defined hierarchical family relationship, which leads to a strong sense of familial self rather than individualized self ^39, 42– 44^. In Sinha *et al*.’s 2002 study on collectivist-individualist intentions and behavior, more than 60% of Indian participants faced with decisions involving their immediate family responded with collectivist behaviors supported by collectivist intentions ^45^. However, when participants faced decisions involving their larger community only 38% responded with purely collectivist behaviors and intentions ^45^. Thus, the familial social group evokes the strongest collectivist response among Indians, while more distal social groups are less likely to evoke a purely collectivist response and are more likely to elicit a mixture of collectivist and individualist intentions and behaviors.

Since vaccines protect both the individual and the larger community, both individualism and collectivistic attitudes may be relevant in understanding the effectiveness of vaccine communication. Collectivist intentions can decrease vaccine hesitancy and increase peoples’ willingness to get vaccinated. The importance of the group in collectivist societies leads to more consideration of others in decisions, including whether to be vaccinated ^46^. One reason for this is that collectivistic intentions are positively correlated with empathy and in a more empathetic society people are more likely to adopt key health precautions such as handwashing and vaccination in response to an increased perception of an infectious disease ^47, 48^. Emphasizing community benefits such as herd immunity in public health communication can increase COVID-19 vaccination in more individualistic cultures where people tend to focus on the benefits to the individual more than the group ^46^. Using data from the COVID-19 Beliefs, Behaviors, and Norms Survey, researchers compared vaccine uptake across 50 countries and found that vaccine intentions were higher in countries with more collectivist cultures and that individuals who endorsed collectivistic values were more likely to accept a COVID-19 vaccine ^49^. The extent to which this approach also applies to vaccination in India is unknown and is the subject of inquiry in this project. 

## Role of protagonist gender

Finally, from a practical perspective, vaccine promotion campaigns also need to make decisions about the kinds of sources they will feature. It is not uncommon to promote vaccination messages through the use of medical spokespersons, public health officials, or credible public health agencies like the Centers for Disease Control and Prevention and WHO ^50, 51^. The source of vaccine information has been strongly linked with trust in that information ^52^. Indeed, decades of communication research have found that information communicated by credible spokespersons are more persuasive for behavior change ^53^. Health communication research tends to focus on source credibility stemming from a spokesperson’s perceived competence and character, but sociodemographic factors – including a source’s race/ethnicity, gender, and social status -- can also have a significant impact on credibility ^54, 55^. Further, it is likely that concordance or homophily between a message’s source and audience can increase receptivity to health messages ^56, 57^. These effects vary by population, health topic, medium, and context.

With the COVID-19 pandemic, a new wave of research focuses on the impact of different spokespersons promoting vaccines – particularly to vaccine-hesitant communities ^58^. Much of this research is focused on US-based and European audiences, with fewer studies exploring messaging effects in low- and middle-income countries ^51, 59^. While concordance between spokesperson and audience is likely important for message tailoring, the culturally specific nature of gender roles and health messaging norms means that each context is likely unique.

India is a patriarchally-oriented society, but health decisions often fall to women ^60^. Women are also more likely to hold community health worker roles (including the all-women ASHA program) that are focused on promoting vaccines ^61^. While these factors may increase the source credibility of a female vaccine advocate, past research has shown that gendered norms may prevent women in Odisha from being seen as autonomous decisionmakers ^62^. The context of COVID-19 vaccine decisions may be unique. A qualitative study from Nair *et al*. found that in Kerala India, men were more likely to be online and therefore more likely to consume COVID-19 vaccine-related messaging ^63^. While younger generations are challenging existing gender norms, for this audience, it isn’t known if a young male or a young female protagonist would be viewed as a more credible advocate for COVID-19 vaccination – or if gender is even relevant in this context. 

## Methods

### Institutional review board statement

The study was conducted in accordance with the Declaration of Helsinki and approved by the Institutional Review Board at The Johns Hopkins University (IRB00018543), 1/23/2022 and Sigma IRB (10075/IRB/21-22) in India on 12/20/2021.

### Study setting

This research was conducted in the eastern Indian state of Odisha, with a population of about 46 million people ^64^. In 2011, 83.3% of the state lived in rural areas, and 16.7% of the population lived in urban areas ^65^. Additionally, 40% of the state’s population belong to Scheduled Tribes (22.85%) or Scheduled Castes (17.13%) ^65^. Odisha is also a state with relatively high levels of childhood immunization. Between 2019 and 2021, 91% of children between 12-23 months received all basic vaccinations against tuberculosis, diphtheria, pertussis, tetanus, polio, and measles and 98% of children between 12-23 months were at least partially vaccinated against the six major childhood illnesses ^64^. 

There have been 1 million confirmed cases and 8,400 deaths reported since the start of the COVID-19 pandemic in Odisha ^66^. Vaccine distribution in the country is decentralized, with each Indian state responsible for local administration and vaccination rates vary widely by state ^67^. As of February 2022, 95% of individuals over the age of 18 years had received the first dose, 92% were eligible for a second dose, and 80.3% of all eligible individuals had received both doses, making vaccination rates in Odisha higher than the national average (Express News Service, 2022). 

### Research team

The research was conducted through a partnership between The Bloomberg School of Public Health, The Johns Hopkins University in the United States and D-COR Consulting in Odisha, India.

### Study design

An online experiment was conducted through a 2 (appeal: individualistic or collectivistic) x 2 (tone: humor or non-humor) x 2 (protagonist gender: male or female) between-subjects design. Participants were recruited through a link posted on the Swasthya Plus Network (SPN) Odia website, and was shared by SPN on their public Odia WhatsApp channels. In order to be eligible for the study, participants needed to be between the ages of 18-35, and speak Odia. We did not check for language ability as the recruitment pages, channel, survey, and videos were all in Odia. Odia-speaking participants (N = 2,700) between the ages of 18–35 years were randomly assigned to watch one of the eight study videos, providing their vaccination attitudes, beliefs, and intentions both before and after viewing the videos. We tested for main effects of appeal, tone, and protagonist gender (as well as the underlying two-way and three-way interactions). During script development, we also sought the feedback from a community advisory board for cultural appropriateness. Videos were shot in December 2021 and the experiment itself was launched in January 2022.

### Video scriptwriting

Many vaccination messages put out by the Indian government use a public service announcement structure in which people are urged to get vaccinated in order to ward off the disease and derive positive benefits, with a primary messaging strategy built around providing accurate vaccine information ^68^. In this project, we adopted a different approach so that our videos would stand out from the din of government-produced announcements. Borrowing ideas from entertainment education ^69^, we chose to depict a common social event (e.g., a child’s birthday) in which vaccination issues would be brought up in an incidental manner, not as the primary thrust of the communication.

The creative team identified various real-world mixed-gender, mixed-age settings that would fit these criteria. Finally, a private social occasion of the birthday of a young family member was selected to be the setting for the video. Within this broad setting, we manipulated appeal, approach, and gender to create eight videos that were identical, except in the variables that were being manipulated. The creative team in Odisha developed the scripts in Odia, the language the videos were filmed and produced in. English translations were used for discussions and feedback with the larger group, which consisted of health communication experts and a former Hollywood scriptwriter with expertise in storytelling. This approach to develop in Odia first, instead of transforming an English script to the language, improved the likelihood that the videos would be culturally relevant.

### Pilot-testing and finalization of the script

The manipulations were scripted into the dialogue across the eight videos in a way to ensure that the videos remained as close in length as possible to one another. Total time for each dialogue block and total number and lengths of different dialogue blocks were kept similar to ensure that there was minimal variance in length of videos.

Before the finalization of the script, the local team in India assembled a community advisory board of 11 individuals, representing diverse perspectives in the Odisha region. The majority of the advisory board members were young adults (under 35) and worked both inside and outside of healthcare. This included two young married couples with children, a doctor, a school principal, an accredited social health activist worker, and a journalist, among others. The group was evenly split between male and female participants. Board members were invited to provide feedback on video scripts. Audio-only versions were recorded for pretesting purposes only. Using a female narrator, four scripts were recorded: two humorous and two neutral, each featuring one individualistic and one collectivistic viewpoint. Board members were interviewed in one-on-one (or pairs for married couples) sessions over Zoom for a total of 10 interviews. Participants were able to re-listen to recordings upon request. Feedback was generally quite positive, with only minor edits suggested. Participants described the scripts as well-done overall, and generally helpful information for individuals who remain unvaccinated. The humorous videos were well received. Participants noted that the humor was used delicately, with a light touch, an important consideration given the seriousness of the subject matter and how many in the community had experienced loss related to COVID-19. Participants could also confidently detect the differences between the individualistic and collectivistic focuses – but did not say which they felt was more effective. The scripts were edited to reflect this feedback before being sent to the filming team.

### Video pre-production

***Camera & production crew***

A professional production team was engaged, consisting of a national-award winning filmmaker, an assistant director, a director of photography, and other crew members. Production logistics were designed to ensure that all the shots would look similar, which meant that the set décor, props, clothing and look etc. would be similar across the multiple days of shooting. A Black Magic Ursa MiniPro 4.6k camera with CP3 lenses was used to film the videos. The Final Cut Pro X was used for editing and the Davinci Resolve 17 was used for color grading on a MacOS.

***Casting***

For the role of the protagonist, versatile actors able to act in non-humorous as well as humorous roles were selected through auditions. Other actors were recruited based on earlier roles they had portrayed in various media productions. The cast members also understood the scientific aspects of the project, such as the need for reducing improvising in acting and to remain aligned with the script.

***Location selection.***

Various locations were scouted, before deciding on the final location, which was a house in a village near Puri, Odisha, which reflected a rural/ semi-urban setting. The location fit well in the story and was also visually appealing.

### Video production approach

 Scholarly programs have isolated and identified different ways that film and video formal features can affect viewing audiences. Formal features in film and video are defined as specific production techniques that are independent of content, message, or story. These features include cuts, dissolves, fades, zooms, angles, sound effects, and more. We sought to keep formal features constant across the different videos, based on findings about how they can have significant effects on viewer experiences ^70– 73^. The entire filming for the eight videos was divided into shots, and similar shots from across all videos were filmed together to ensure that all of them had the same camera angle, focal lengths, pans, zooms, and backdrop weather conditions to align across all eight scripts.

### Post-production editing

 The editing process began with the selection and cataloging of shots as per the various scripts. The first video was created with all the shots and their sequences in the video being documented. The remaining videos were created by using the first video as a template and changing the specific shots as necessary. Put another way, we did not shoot each video from beginning to end before shooting the subsequent video. Rather, we kept the camera focused in a particular way and then shot all eight scenes pertaining to that camera angle before moving on to the next scene. A sample of the shot list for the template video is shown in Table 1 which was used as a guide to ensure consistency across the eight videos. Each video was subsequently reviewed by a member of the creative team well versed with the research needs and aims, to ensure similarity as well as accuracy in the variations. This post-production editing can be a complex process due to a high volume of seemingly similar but different shots, especially if the creative team members are not familiar with this approach of making videos.

**Table 1.** Description of shot list sample from the template video.

Video	Start	Stop	Camera	Scene
**Template**	0.00	0.03	Close up	Cake being cut
	0.04	0.07	Close up	Helmet on Table
	0.08	0.11	Wide	Munna (the male protagonist) being greeted
	0.12	0.15	Two shots	Handing cake

### Dissemination

 After conducting the online experiment, we selected two of the eight videos for dissemination so that we could also study the comments they would generate from viewers and conduct a social network analysis of the viewers themselves. We chose only two videos (and not all eight) so as to minimize perceptions of repetition by the audience. We selected the female, individualistic, non-humor video and then the diametrically opposite one that differed on all three variables (hence a male, collectivistic, humor video). Dissemination was done on the Swasthya Plus Network (SPN) Odia website, Facebook page, and YouTube channel ^74^. The videos were given titles, descriptions, and thumbnails to generate curiosity and interest. A conscious effort was made to ensure the videos did not resemble vaccine public service announcements, given the perceived fatigue among the audience toward such messaging. The videos were disseminated across both platforms, with an intentional posting gap between the release of the first and second video. The video links were then shared with tens of thousands of people connected with Swasthya Plus and partners via WhatsApp.

### Evaluation of videos

Videos will be evaluated in 3 ways:

1. Online experiment,
2. Qualitative investigation via focus groups/ interviews,
3. Social media analysis.

***Online experiment***

The online experiment was a survey questionnaire hosted on the Qualtrics platform with four parts: consent, pre-exposure questions, video randomization, and post-exposure questions. The survey questionnaire was developed specifically for this project, but included scales on general vaccine hesitancy and individualism/collectivism adapted from previously published work by Quinn *et al*. 2019 and Singelis *et al*. 1995, respectively ^10, 42^. Additional questions were added based on the specific context of COVID-19, the target audience of young adults, and the setting of Odisha, India. The final questionnaire was not validated, however all the team members (N=13) pilot tested the questionnaire, and the final version was created taking into account the local setting in Odisha, comments and discussions from the team and being mindful of how long it would take respondents to complete it. The questionnaire can be found as *Extended data* ^75^.

The survey was expected to take each participant 20–30 minutes to complete and participants were compensated on completion. To recruit participants for the online experiment component, our partner Swasthya Plus Network advertised the experiment through a link on their WhatsApp channels. 2,349 responses were collected over a six-day period in February 2022.

**a.   Consent**

Participants who clicked on the experiment link were then taken to the consent page, which asked for the participant’s age to determine eligibility. Although being able to speak Odia was an inclusion criterion, we did not need to ask about their language abilities because the questionnaire was conducted in Odia. If they did not meet the eligibility criteria, they were thanked and dismissed. In the consent form, the structure of the questionnaire and topics were outlined before each section. Additionally, participants were reminded that the questionnaire was voluntary and that they would receive a compensation of ₹200 (Indian Rupees) via google pay or phonePe, both digital wallets.

**b.   Pre-exposure questions**

We started the survey by asking the respondents about their age, gender and their vaccination status. Participants who indicated that they had not received a COVID-19 vaccine were asked what their primary reason was for refraining from getting the particular vaccine. In an effort to understand attitudes and beliefs about COVID-19 vaccines (including vaccine hesitation towards COVID-19), participants were asked to rate their agreement on a series of statements adapted from Quinn *et al*. (2019) on a five-point Likert scale from “strongly disagree” to “strongly agree” ^10^. Additionally, we asked how often and how likely specific COVID-19 thoughts had entered their minds in the last three months including the likelihood of contracting COVID-19 and the frequency that they thought about COVID-19 vaccinations. Finally, we included 13 questions about collectivism and individualism from Singelis *et al*.’s 1995 vertical and horizontal collectivism and individualism scale ^42^. The three statements of each type of collectivism/individualism with the highest factor loadings along with one very culturally appropriate statement about family was included in the questionnaire.

**c.   Video randomization**

Subsequently, respondents were randomized to view one of the eight videos that were identically shot and edited with the exception of the independent test variables. The eight videos were categorized as follows:

· Female/Collective and Humorous,

· Female/Collective and non-humorous,

· Female/Individual and Non-humorous,

· Female/Individual and Humorous,

· Male/ Collective and humorous,

· Male/Collective and Non-humorous,

· Male/Individual and Humorous,

· Male/Individual and Non-humorous.

 After, watching the videos participants were asked how many times they had watched the videos and the duration for which they watched the video to assess whether they had completed watching the video.

**d.   Post-exposure questions**

After being randomized to view one of the videos, participants were asked about their reactions, including the extent to which they found the video to be important, or relevant or caused them to reflect about the COVID-19 vaccine. Participants were also asked about the tone of the video as a manipulation check to gauge whether the humorous videos were perceived as humorous, whether the collectivistically oriented videos were perceived as such, and the extent to which viewers correctly remembered the protagonist gender. We also asked questions about the characters in the video to assess recollection. This included items on source credibility adapted from McCroskey & Teven (1999) ^76^. Lastly, we collected information about participants’ social media use, followed by their general demographic information.

***Qualitative component***

Qualitative research will focus on eliciting the reactions from vaccine-hesitant individuals, identified through the online survey. Our local partners at D-COR will conduct 12 online focus groups, to discuss participant's beliefs and reactions to the videos. Focus groups will be stratified by both age and gender. Of the pool of participants who agree to participate in the qualitative work, eligibility will be decided based on their age and gender (to get a sound distribution of age and gender groups). Focus groups will run for 90 minutes and be conducted in Odia. In the first half of the focus group, moderators will guide discussion around beliefs and attitudes around vaccines – both COVID-19 and vaccines in general – and invite participants to share their concerns. In the second half of the focus groups, participants will be invited to share their reactions to the videos.

 We also plan to conduct up to 20 semi-structured interviews with individuals who may prefer the one-on-one format. This also provides more flexibility for the research team to delve deeper into any emergent themes that may arise during focus groups.

***Social media analysis component.***

Two diametric vaccine videos will be posted on YouTube and promoted through social media channels, including Facebook and WhatsApp. Engagement with videos will be tracked through YouTube analytic metrics. All public comments posted under the videos in English, translated from Odia when required will be analyzed for recurring keywords, emerging themes, and sentiment, using tools from YouTube Data Tools, TextBlob and Vadar on Communalytic. Additionally, social network analysis to visualize the comment network for each video as well as the broader channel network to assess engagement across all videos will be conducted. Gephi software will be used to construct networks, with measures including the number of nodes (individuals), edges (connections between nodes), degrees (measure of interaction), and modularity (measure of communities within the network).

## Measurement

### Statistical analysis and power calculation

**Online experiment component:** We assumed that, prior to exposure, 30% of the population would be vaccine-hesitant and that exposure would affect the attitudes of 10% of the viewers. To achieve power of 80%, with an alpha of 5%, the required sample size would be 294 per group, which we rounded up to 300. To be able to conduct sub-analyses along caste (three subgroups) and three age groups (i.e., nine groups in total), the overall required sample size was calculated to be 2,700. 

**Qualitative component:** From the survey respondents ( *n*=2,700), we will identify the 10% ( *n*=270) with greatest vaccine hesitancy, as determined through their pre-exposure scores on the vaccine confidence index. This subsample will be invited to participate in focus groups. From this pool, we will purposively select participants to fill up to 12 focus groups, each with 8 participants. To ensure we hear diverse viewpoints from different segments of the population, groups will be stratified by both age (18–22, 23–30, 31–45) and gender (male, female). Following this design, we expect a maximum of 100 participants ( Table 2).

**Table 2.** Expected numbers and description of focus group (FG) participants.

	Male	Female	Total
Age 1: 18–22 years	2 FGs ( *n*=16)	2 FGs ( *n*=16)	4 FGs ( *n*=32)
Age 2: 23–30 years	2 FGs ( *n*=16)	2 FGs ( *n*=16)	4 FGs ( *n*=32)
Age 3: 31–45 years	2 FGs ( *n*=16)	2 FGs ( *n*=16)	4 FGs ( *n*=32)
Total	6 FGs ( *n* =48)	6 FGs ( *n* =48)	12 FGs ( *n*= 96)

Additionally, time and resources have been allocated to conduct semi-structured online interviews with up to 20 additional people to delve deeper on any emergent topics that may arise over the course of the study. This would lead to a maximum of 120 participants in the qualitative portion of the study.

## Results

Ultimately, findings from this work will provide guidance to health communication professionals on how to frame their vaccination messages regarding whether appealing to the collective good or the individual benefit. In addition, when and how to use humor, and how male and female protagonists garner different reactions among different audiences will be determined. We also want to understand how these factors may work synergistically. The overall goal is to explore which combinations of messaging features have the greatest impact to reduce vaccine hesitancy and boost confidence in vaccination. Through the qualitative analysis, we have the opportunity to gain a deeper understanding of these specific message features and their relevance to the vaccine-hesitant in Odisha. Through the social network analysis, we will explore the connections between users and gain insight into information-seeking behaviors around vaccines. While this project is narrowly focused on COVID-19 vaccine, we anticipate that valuable insights gleamed from this work will have important implications for messages on vaccination for other diseases and elucidate how to craft and disseminate messages through brief videos on the Internet.

## Study status

At this stage, the creation and dissemination of the videos has occurred. The online experiment component the survey questionnaire and the social media analysis has begun. The follow-up qualitative work and analysis have yet to happen. We are currently testing the expansion of the social media analysis to include A/B testing on Facebook.

## Data availability

### Underlying data

No underlying data are associated with this article.

### Extended data

Open Science Framework: Creation, dissemination, and evaluation of videos to promote COVID - 19 vaccination in India. https://doi.org/10.17605/OSF.IO/4AYPV ^75^.

This project contains the following extended data:

- VCF Questionnaire Code Book.csv

Data are available under the terms of the Creative Commons Zero "No rights reserved" data waiver (CC0 1.0 Public domain dedication).
