# Supplementary material for: Creation, dissemination, and evaluation of videos to promote COVID-19 vaccination in India: A research protocol
Source: Gates Open Res. 2022 Oct 26;6:82. Originally published 2022 Jul 25. [Version 2] doi: 10.12688/gatesopenres.13628.2 (PMC9652135; doi:10.12688/gatesopenres.13628.2)
Supplement: Peer review history

# Peer review: Reviewer response for version 2

**DOI:** 10.21956/gatesopenres.15415.r32711

**Published:** 2022-11-11

**Authors:** Shaffi Fazaludeen Koya

**Affiliations:** 1 Department of Epidemiology, Boston University School of Public Health, Boston, Massachusetts, USA

Thank you for your response. 

 My suggestion on sampling was aimed at addressing the under-representation of particular vulnerable groups. However, in the light of the constraints explained in your response, I agree with your methods. Thank you.

Is the study design appropriate for the research question?

Partly

Is the rationale for, and objectives of, the study clearly described?

Yes

Are sufficient details of the methods provided to allow replication by others?

Partly

Are the datasets clearly presented in a useable and accessible format?

Not applicable

Reviewer Expertise:

infectious disease epidemiology, health policy, health systems, evaluation methods, and antimicrobial policies.

I confirm that I have read this submission and believe that I have an appropriate level of expertise to confirm that it is of an acceptable scientific standard.

# Peer review: Reviewer response for version 1

**DOI:** 10.21956/gatesopenres.14905.r32412

**Published:** 2022-09-09

**Authors:** Md. Aminul Islam

**Affiliations:** 1 Department of Media Studies and Journalism, University of Liberal Arts Bangladesh (ULAB), Dhaka, Bangladesh

The authors investigated the role of videos containing humor appeal in promoting vaccine acceptance in the context of India. It is a timely work. Communicating public health issues is a big global challenge today. This paper would contribute to literature on message design and communication on public health communication, especially vaccine uptake in the context of non-western countries. I found that the authors have used appropriate methods to deal with issues. Considering the merit of the work, novelty of findings and methodological rigors, I recommend to accept the paper for indexing.

Is the study design appropriate for the research question?

Yes

Is the rationale for, and objectives of, the study clearly described?

Yes

Are sufficient details of the methods provided to allow replication by others?

Yes

Are the datasets clearly presented in a useable and accessible format?

Yes

Reviewer Expertise:

Communication in healthcare; Public health communication; Social determinant of health; and Social support network and mental health

I confirm that I have read this submission and believe that I have an appropriate level of expertise to confirm that it is of an acceptable scientific standard.

# Peer review: Reviewer response for version 1

**DOI:** 10.21956/gatesopenres.14905.r32322

**Published:** 2022-08-02

**Authors:** Shaffi Fazaludeen Koya

**Affiliations:** 1 Department of Epidemiology, Boston University School of Public Health, Boston, Massachusetts, USA

Thank you for the opportunity to review the study protocol, "Creation, dissemination, and evaluation of videos to promote COVID-19 vaccination in India: A research protocol".

 This is a very relevant area of research and the authors have proposed a very interesting study. I would like to congratulate the research team on their innovative approach.

 My comments are aimed at helping to clarify some of the methodological details, as this study could be very insightful if it generates more generalizable data. This is all the more so as the study is an opt-in design using online platforms. Also, a more detailed description of the analysis plan would be helpful.

  

1. The COVID 1st dose vaccination rate in the state is already very high. This means that a large proportion of your study respondents will already be vaccinated. You may consider methods to get an adequate number of respondents who are unvaccinated. Additionally, you may also consider comparing participants who received one dose vs. those who received two doses. This is important as the contexts in which the 1st dose and 2nd dose were distributed were different. 
2. The vaccination rate among socially excluded groups is relatively low in India as opposed to elsewhere. In this circumstance, you may want to take measures to have an adequate representation of such social categories in your study. This is important considering the reported higher vaccine hesitancy in certain groups.
3. I would suggest using iterative proportional fitting (raking) or propensity weighting or a combination of methods to achieve the above objectives.
4. Try to include details of the proposed quantitative analysis of the online experiment data.

Is the study design appropriate for the research question?

Partly

Is the rationale for, and objectives of, the study clearly described?

Yes

Are sufficient details of the methods provided to allow replication by others?

Partly

Are the datasets clearly presented in a useable and accessible format?

Not applicable

Reviewer Expertise:

infectious disease epidemiology, health policy, health systems, evaluation methods, and antimicrobial policies.

I confirm that I have read this submission and believe that I have an appropriate level of expertise to confirm that it is of an acceptable scientific standard, however I have significant reservations, as outlined above.

# Response

**Published:** 2022-10-18

**Authors:** Ananya Bhaktaram

**Affiliations:** Johns Hopkins Bloomberg School of Public Health, USA

Dear Dr. Koya,

 Thank you for taking the time to read our manuscript and for your thoughtful comments. Here are our responses/corresponding edits.

1. Your concerns about the sample population due to the high vaccination rates in Odisha are valid. The application for the grant that supported this work was submitted before COVID-19 vaccinations had moved out of the testing and development phase, and prior to roll-out. Once we received funding COVID-19 vaccines had already become widely available in India and the issues surrounding COVID-19 vaccination behavior became ones of availability and not hesitancy. Focusing on behaviors would have resulted in a terribly skewed sample.  Thus, we chose to pivot the focus of our research to look at peoples’ attitudes toward vaccination rather than vaccination itself.
2. You bring up a valid point that vaccination rates are significantly lower in socially excluded groups. However, due to the initial constraints and conception of this study we did not focus on the differences between tribal versus non-tribal groups and so we didn’t strategically over sample. Additionally, during the timeframe in which the study was conducted, little was known about people’s attitudes toward COVID-19 in general, which is why we did not focus on underserved populations. However, we will look and stratify across different demographic groups for our main effects paper.
3. We respect your comments about using iterative proportional fitting/propensity weighting in our methods. Indeed, this would be the preferred method if we had an observational study in which we would need to create equivalent groups. In our study, however, we have a randomized design in which people were assigned, at random, to particular experimental arms, which that the various arms of the study, by design, are equivalent. We hope we are not misreading the reviewer’s comments. 
4. In the latest edition of the manuscript, we have included additional details of the proposed quantitative analysis of the online experimental data.
